# Projected hydroclimatic changes in Xinjiang under bias-corrected CMIP6 scenarios

**DOI:** 10.3389/fpls.2025.1679735

**Published:** 2025-12-09

**Authors:** Yang Xu, Shenzhen Tian, Xueping Cong, Mengxin Bai, Juncheng Zhang

**Affiliations:** 1 School of Geography, Liaoning Normal University, Dalian, Liaoning, China; 2 Center for Human Settlements, Liaoning Normal University, Dalian, Liaoning, China; 3 Research Base of Urban Agglomeration in Central-South Liaoning of China Urban Agglomeration Research Base Alliance, Liaoning Normal University, Dalian, Liaoning, China; 4 Liaoning Research Base for Synergistic Development of Human Settlements and Talents, Dalian, Liaoning, China; 5 Beijing Municipal Climate Center, Beijing Meteorological Service, Beijing, China; 6 Meteorological Bureau of Dashiqiao, Yingkou, China

**Keywords:** CMIP6, BCCAQ, Xinjiang, climate change, drought, SPEI

## Abstract

Understanding future hydroclimatic variability in arid regions is essential for sustainable development and climate adaptation. This study uses bias-corrected CMIP6 daily climate projections, derived by applying the BCCAQ method to ERA5 reanalysis and surface station data, to investigate the spatiotemporal evolution of key meteorological variables and drought conditions over Xinjiang during 2031–2060 under three SSP scenarios (SSP1-2.6, SSP3-7.0, and SSP5-8.5). Results reveal significant warming trends across all scenarios, with stronger increases under high-emission pathways (up to 0.76°C/10a under SSP585), accompanied by enhanced potential evapotranspiration (PET) and widespread aridification. While precipitation shows an upward trend under SSP370 and SSP585, the warming-induced evaporative demand dominates, particularly in southern Xinjiang and the eastern basins. The SPEI index indicates an intensifying drought risk, with spatial patterns characterized by a “dry south–wet north” gradient and stronger basin aridification relative to mountainous regions. Moreover, this study highlights the physical mechanism linking temperature rise, enhanced PET, and intensified drought, providing robust empirical evidence for regional climate risk assessment and adaptation strategies in Central Asia. Despite methodological advantages, limitations associated with spatial resolution and structural uncertainty of GCMs persist, suggesting the need for integrating regional climate models (RCMs) and extreme event analyses in future research.

## 1 Introduction

Against the backdrop of global warming, extreme climate events are occurring with greater frequency and intensity, and phenomena such as droughts, floods, and heatwaves have increasingly emerged as key drivers impacting agriculture, ecosystems, and societal progress (IPCC et al., 2021; Bewket et al., 2022; Guan et al., 2022; Xu et al., 2022; Ma et al., 2023; Torres-Vazquez et al., 2024). Particularly in arid and semi-arid regions with complex terrain, the climate system exhibits heightened sensitivity to external disturbances, and the uncertainty of future climate change is also greater (Yang et al., 2021; Guo et al., 2023). Consequently, enhancing the precision of future climate change modeling in these regions has emerged as a key focus of contemporary climate science and ecological agriculture studies.

Xinjiang is located in the northwest of China, in the heart of the Eurasian continent, and is characterized as a typical arid and semi-arid region (Yao et al., 2022). Mountain ranges and basins are interlaced throughout the region, with limited precipitation, strong evaporation, and a markedly uneven spatiotemporal distribution of water and heat resources. In recent years, Xinjiang has experienced a significant rise in temperature, changes in the spatiotemporal patterns of precipitation, and complex trends in key meteorological variables such as wind speed, relative humidity, and radiation (Deng et al., 2022; Xu Y. et al., 2024). Such indicators of climate change have profoundly influenced agricultural water requirements, crop phenology, and the functioning of regional ecological processes.

Variations in wetness and dryness serve as key indicators of regional water balance, directly linked to crop water stress, growth cycles, biomass accumulation, and yield stability. Constructing and applying drought indices provide a fundamental foundation for research on moisture variability (Yuan et al., 2025). Among them, the Standardized Precipitation Evapotranspiration Index (SPEI), which simultaneously accounts for precipitation and potential evapotranspiration (PET), can comprehensively characterize drought formation mechanisms and temporal evolution, and has been widely applied to drought studies globally and in China (Adam, 1998; Huang et al., 2017; Yao et al., 2018; Yao et al., 2021; Deng et al., 2022; Wang et al., 2022; Yao et al., 2022; Li et al., 2023). Research based on SPEI has preliminarily revealed the spatiotemporal characteristics of wet-dry evolution and its main driving factors during historical periods in Xinjiang (Deng et al., 2022; Xu Y. et al., 2024). However, under the context of synergistic changes in various climatic factors and regional response differences, significant uncertainties remain in the trends of wet-dry changes. Therefore, clarifying the future evolution pathways of SPEI and its dominant meteorological factors is of great practical significance for improving the prediction capacity of agricultural climate risks in arid regions, optimizing crop planting structures, and enhancing ecosystem adaptation strategies.

At present, future climate projections predominantly rely on the Coupled Model Intercomparison Project Phase 6 (CMIP6), which provides multi-model ensemble simulations under various Shared Socioeconomic Pathways (SSPs) and has established a fundamental framework for advancing climate research (Eyring et al., 2016; Riahi et al., 2017). Earlier studies have demonstrated these applications: for instance, Cao et al. (2023), drawing on 30 CMIP6 global climate models, systematically assessed hydroclimatic trends in arid Central Asia and concluded that enhanced potential evapotranspiration will intensify both the frequency and severity of droughts across Xinjiang and adjacent regions (Cao et al., 2023). Complementarily, other analyses have revealed that extreme events—such as heatwaves and heavy precipitation—are projected to escalate under SSP585, thereby exacerbating the vulnerability of regional ecosystems (Guan et al., 2022). More recent investigations have reinforced and substantiated the aggravating trajectory of climate risks in this region. Wu et al. (2025) employed attribution analysis and risk projections to demonstrate that, under high-emission scenarios, the frequency and persistence of hydrological droughts in Central Asia will rise markedly (Wu et al., 2025). Sun et al. (2025), analyzing a long-term dataset spanning 1948–2022, revealed a coherent intensification across multiple drought types and identified their dominant climatic drivers (Sun et al., 2025). Furthermore, Li et al. (2025) projected that the Ili River Basin—closely linked to Xinjiang—will experience heightened comprehensive drought risks by mid-century (Li et al., 2025).Nevertheless, the raw outputs of CMIP6 remain constrained by their coarse spatial resolution, which hampers the accurate representation of local climate processes in Xinjiang’s complex terrain. This limitation is particularly pronounced for critical variables such as extreme precipitation and drought frequency, where systematic biases continue to persist (Xu et al., 2021; Xu Z. et al., 2024; Zhang Y. et al., 2024).

The Bias Correction Constructed Analogues with Quantile mapping reordering (BCCAQ) method integrates the Constructed Analogues (BCCA), Bias Correction Climate Imprint (BCCI), and Quantile Mapping (QDM) approaches, effectively reducing systematic errors while preserving the structure of climate time series (Gebrechorkos et al., 2023). Previous research indicates that BCCAQ markedly enhances the accuracy of simulations for critical climate variables—including temperature, precipitation, evapotranspiration, and drought—making it especially effective for areas with complex topography and diverse climatic conditions (Rettie et al., 2023; Yang and Tang, 2023).

In this study, a high-resolution reanalysis dataset of Xinjiang is employed as the reference benchmark, and the BCCAQ method is applied to perform daily bias correction on the ensemble mean of ten CMIP6 global climate models. Building upon this bias-corrected framework, we conduct a systematic assessment of the spatiotemporal evolution of key meteorological variables—including temperature, precipitation, wind speed, relative humidity, downward shortwave radiation, and potential evapotranspiration—during 2031–2060, thereby elucidating the regional signatures of future dry–wet variability under different emission scenarios. To our knowledge, this work represents the first systematic application of BCCAQ in the arid zone of Xinjiang, significantly improving the fidelity of climate simulations over regions with complex topography. By integrating multiple meteorological variables into the assessment of the SPEI, this study moves beyond precipitation-centered analyses and provides new insights into the mechanisms governing future hydroclimatic variability. Collectively, these findings not only establish a robust climatic foundation for agricultural development, water resource management, and vegetation adaptation in Xinjiang’s arid regions, but also deliver essential data support and scientific evidence for broader applications in botany and agricultural ecology.

## 2 Data and methodology

### 2.1 Study area description

**Figure 1:**
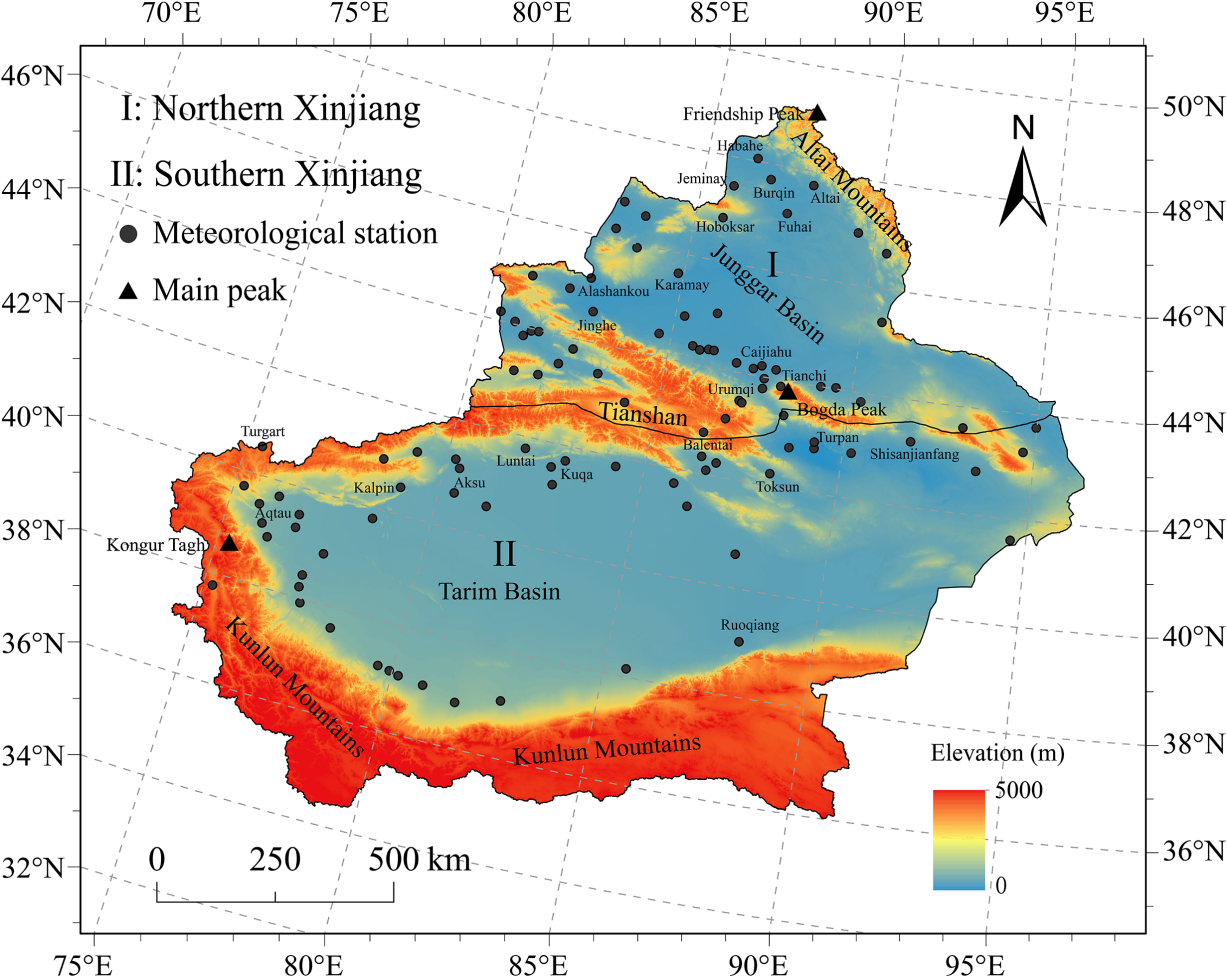
Overview of the study area and distribution of meteorological stations.

Situated in northwestern China and at the core of the Eurasian continent, Xinjiang is characterized as an arid and semi-arid region with a fragile ecosystem and high sensitivity to climate change, and has become one of the crucial regions for global climate change studies. Topographically, Xinjiang exhibits a “three mountain ranges separated by two basins” pattern, with the Tianshan Mountains running across the center (**Figure1**), dividing the region into two climatic subregions (Zheng et al., 2013; Xu Y. et al., 2024): Northern Xinjiang (I) and Southern Xinjiang (II). Northern Xinjiang belongs to the mid-temperate zone, with an average annual temperature of 5.9°C and precipitation of 253.0 mm, mainly covering the northern slope of the Tianshan Mountains, the Junggar Basin, and the Ili Valley; Southern Xinjiang belongs to the warm temperate zone, with an average annual temperature of 10.5°C and precipitation of 68.5 mm, encompassing regions such as the southern slope of the Tianshan Mountains, Turpan, and the Tarim Basin, where the climate is hot and arid with very little rainfall and pronounced drought characteristics.

### 2.2 Data description

#### 2.2.1 Reference data

The meteorological observation data are derived from the China National Ground Meteorological Station Daily Meteorological Elements Dataset (V3.0), which includes daily observations from 105 stations in Xinjiang during 1961–2020 (https://data.cma.cn/). The observed variables include maximum temperature, minimum temperature, mean temperature, precipitation, mean air pressure, mean wind speed, relative humidity, and sunshine duration. Specifically, temperature, pressure, wind speed, and relative humidity are measured at four daily intervals (02, 08, 14, and 20 h), whereas precipitation is observed over two periods each day (20–08 h and 08–20 h).

The ERA5 reanalysis data used in this study include both surface and pressure-level fields at a spatial resolution of 0.25° × 0.25°. The surface variables comprise 2-m air temperature, precipitation, surface pressure, mean sea-level pressure, 10-m wind speed, and soil temperature and moisture. The pressure-level data provide wind (u and v components), temperature, geopotential height, and relative humidity at 37 levels from 1000 to 1 hPa.

#### 2.2.2 Climate model data

The climate simulations analyzed in this study (1961–2060) were obtained from ten CMIP6 models, namely CNRM-CM6-1, CNRM-ESM2-1, CanESM5, EC-Earth3, GFDL-ESM4, IPSL-CM6A-LR, MIROC6, MPI-ESM1-2-HR, MRI-ESM2-0, and UKESM1-0-LL (https://data.isimip.org/). These models were selected because they provide continuous daily meteorological variables for the entire study period, thereby ensuring the feasibility of day-scale analyses required in this work. Since the native spatial resolutions of these models differ, the Inter-Sectoral Impact Model Intercomparison Project (ISIMIP) performed a standardized bias correction, adjusting model–observation discrepancies in monthly climatology while preserving both the long-term variability and the absolute magnitudes of the simulations (Lange, 2019). The bias-corrected datasets demonstrate notable improvements in both accuracy and internal consistency, rendering them more robust for regional-scale climate impact assessments. Accordingly, this study employed bias-corrected daily datasets at a 0.5° × 0.5° resolution to construct and analyze near-surface meteorological conditions in Xinjiang under future scenarios.

### 2.3 Methodology

#### 2.3.1 Drought index

In this study, a 3-month step Standardized Precipitation Evapotranspiration Index (SPEI-3, hereafter referred to as SPEI) is employed to analyze the seasonal wet-dry variation characteristics in Xinjiang. This index is derived by calculating the difference between precipitation and potential evapotranspiration, followed by a normal standardization process, and mainly reflects the degree of drought deviation from normal years. The potential evapotranspiration is calculated using the Penman-Monteith equation (Adam, 1998).


$$PET=\frac{0.408\Delta (R_{n}-G)+\gamma \frac{900}{T+273}u_{2}(e_{s}-e_{a})}{\Delta +\gamma (1+0.34u_{2})}$$

In the equation: PET stands for potential evapotranspiration (mm/d); Rn refers to net radiation on the surface of a reference crop (MJ/(m²·d)); G is the soil heat flux (MJ/(m²·d)); γ denotes the psychrometric constant (kPa/°C); Δ is the slope of the saturation vapor pressure curve at a specific temperature (kPa/°C); T represents the daily mean temperature (°C); u2 is the wind speed at 2 m height (m/s); es is the saturation vapor pressure (kPa); and ea is the actual vapor pressure (kPa). The SPEI is computed by evaluating the cumulative precipitation and the difference between precipitation and PET over a 3-month period, fitting the series to a log-logistic probability distribution, and subsequently performing normal standardization of the cumulative probability density (Vicente-Serrano et al., 2010).

#### 2.3.2 Evaluation metrics

Several metrics were selected to evaluate the performance of the data, including Absolute Error (AE), Pearson Correlation Coefficient (CC), and Root Mean Square Error (RMSE), in order to quantitatively assess the accuracy of the dataset. AE represents the systematic bias of the dataset, where negative and positive values indicate underestimation and overestimation, respectively. CC reflects the linear correlation between the dataset and the meteorological station observations. RMSE measures the mean error of the dataset. To quantify the simulation capability of the model under different parameterization scheme combinations, the Distance between Indices of Simulation and Observation (DISO) index, which integrates statistics such as CC, Normalized Root Mean Square Error (NRMSE), and Normalized Absolute Error (NAE), is employed. This index evaluates the simulation accuracy by calculating the distance between the simulated and observed fields in a three-dimensional coordinate system. The formulas for calculating these indices are as follows:


$$AE=\frac{1}{n}\sum_{i=1}^{n}\left(y_{i}-x_{i}\right)$$

$$CC=\frac{\sum_{i=1}^{n}\left(x_{i}-\bar{x}\right)\left(y_{i}-\bar{y}\right)}{\sqrt{\sum_{i=1}^{n}{\left(x_{i}-\bar{x}\right)}^{2}}\sqrt{\sum_{i=1}^{n}{\left(y_{i}-\bar{y}\right)}^{2}}}$$

$$RMSE=\sqrt{\frac{1}{n}\sum_{i=1}^{n}{\left(x_{i}-y_{i}\right)}^{2}}$$

$$NRMSE=\frac{RMSE}{\left|\bar{y}\right|}$$

$$NAE=\frac{AE}{\left|\bar{y}\right|}$$

$$DISO=\sqrt{{\left(CC-1\right)}^{2}+{\left(NRMSE\right)}^{2}+{\left(NAE\right)}^{2}}$$

#### 2.3.3 Bias correction of climate model data

Bias Correction Constructed Analogues with Quantile mapping reordering (BCCAQ) is a hybrid statistical downscaling method, which integrates the Bias Correction Constructed Analogues (BCCA) and Bias Correction Climate Imprint (BCCI) approaches to enhance the spatial resolution of climate data and correct systematic biases (Hunter and Meentemeyer, 2005; Maurer et al., 2010; Cannon et al., 2015; Werner and Cannon, 2016). It performs well in simulating extreme climate events, preserving spatial covariance, and maintaining temporal continuity. BCCA utilizes the Constructed Analogues technique to align climate model outputs with high-resolution reference datasets in order to maintain the statistical properties of the model data. BCCI interpolates the original climate model data to a higher resolution and applies Quantile Delta Mapping (QDM) for bias correction to improve data quality. In the BCCAQ process, the BCCI, BCCA, and QDM algorithms operate independently, and by integrating their outputs, the downscaled data’s ability to reproduce extreme events is enhanced, while spatial covariance and daily sequence characteristics are optimized, making it more applicable to global and regional climate studies. This study uses ERA5 reanalysis data together with surface station observations to bias-correct the ensemble mean of the GCMs, with the detailed procedure described below:

a. Calibration of Historical Data:

We applied a three-dimensional variational (3DVAR) data assimilation system, together with surface station observations, to optimize the initial and boundary conditions of ERA5 over Xinjiang, thereby generating a daily gridded dataset at 10 km spatial resolution for 1961–2014. Historical GCM outputs at coarse resolution (0.5°) were then compared and calibrated against this high-resolution reference grid to diagnose long-term biases. First, the BCCA method was applied to compare and adjust the spatial patterns of GCM fields relative to the reference, correcting their spatial structures. Second, the BCCI method was used to further adjust the BCCA-processed data, ensuring consistency with observations not only in the mean state but also in long-term trends and extremes. Third, the QDM method was applied so that the downscaled data matched the observed probability distribution while preserving the long-term climate change signal projected by the GCMs. Finally, Empirical Copula Coupling (ECC) was employed to ensure that the temporal dependence structure of the downscaled series remained consistent with that of the original GCM data.

b. Application to Future Scenario Data:

The bias correction and distribution mapping relationships derived from the historical period are directly applied to the coarse-resolution model data of future scenarios to generate the corresponding high-resolution data. This process relies on the stationarity assumption, meaning that the bias structure and distribution relationships are assumed to remain stable between the historical and future periods, enabling the application of historical calibrations to future data. For future data, calibration parameters (such as mean bias, variance scaling, and distribution mapping) are not recalculated but are instead used from those obtained during the historical period to correct the future data. BCCA is skipped for the future data, and the procedure begins with BCCI, directly applying the BCCI and QDM relationships established from the historical period to perform identical calibration operations as those used for historical data.

c. Production of Final Output Data:

The high-resolution dataset derived using the BCCAQ method for the historical period (1961–2014) will be adopted as the final output. In the case of future scenarios (2015–2100), historical calibration parameters will be directly employed to generate scenario-specific high-resolution datasets.

The gridded data from 1961 to 2014 are obtained from the Three-Dimensional Variational Xinjiang Meteorological Forcing Dataset (3DVAR-MF-XJ), which has a spatial resolution of 10 km and a temporal resolution of 1 hour (https://www.xjsedata.cn). This dataset is based on 3DVAR technology, which assimilates and optimizes the initial and boundary fields, effectively overcoming the issues of low resolution and large errors in reanalysis data over Xinjiang’s complex terrain, while improving the accuracy of elements such as temperature, wind speed, and precipitation. The meteorological variables include temperature, precipitation, wind speed, relative humidity, radiation, and air pressure.

## 3 Results and analysis

### 3.1 Validation of climate model dataset reliability

Using station observations, we systematically evaluated the performance of the scenario-specific high-resolution datasets in reproducing monthly key meteorological variables, including mean temperature (TAV), precipitation (PRE), wind speed (WIN), relative humidity (RHU), pressure (PRS), and downward shortwave radiation (RSDS).The simulation performance of different models was quantified using four key metrics: correlation coefficient (CC), absolute error (AE), root mean square error (RMSE), and DISO. DISO, which integrates CC, AE, and RMSE, serves as a comprehensive indicator reflecting the overall capability of the models, and the ranking of DISO values was used to assess the superiority of different models.

**Figure 2:**
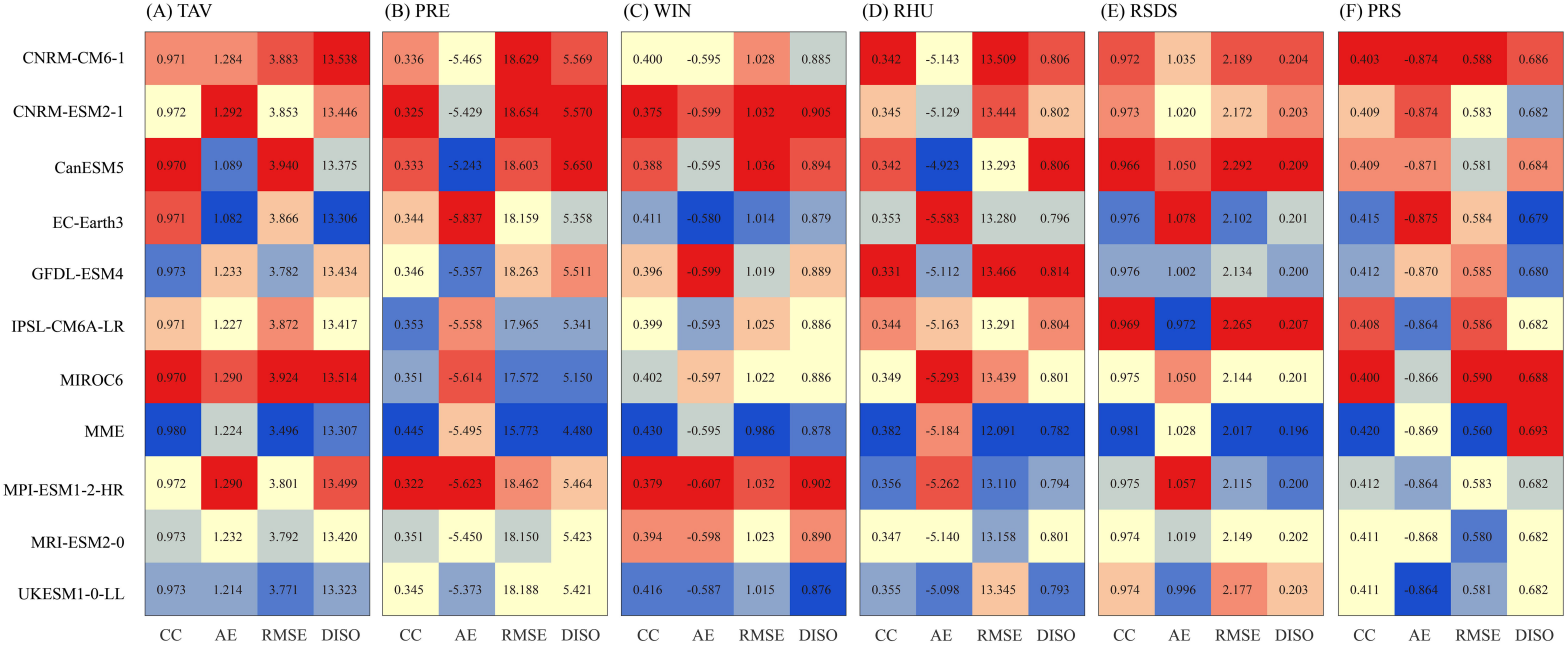
Correlation coefficient (CC), absolute error (AE), root mean square error (RMSE), and DISO values of temperature, precipitation, wind speed, relative humidity, downward shortwave radiation, and surface pressure **(A–F)** from 10 GCMs and the multi-model ensemble mean (MME) compared with station data.

Regarding temperature (TAV) simulation, EC-Earth3 shows the lowest DISO value of 13.306, demonstrating superior overall performance, while CNRM-CM6-1 (DISO = 13.538) and MIROC6 (DISO = 13.514) exhibit higher DISO values, indicating larger systematic biases in temperature simulation (**Figure 2**). The DISO value of the multi-model ensemble (MME) is 13.307, ranking just behind EC-Earth3, suggesting superior ability in temperature simulation. For precipitation (PRE) simulation, the MME has the lowest DISO value of 4.480, indicating higher overall quality in precipitation simulation, while CanESM5 has the highest DISO value of 5.650, suggesting larger precipitation simulation errors. Additionally, MIROC6 (DISO = 5.150) and IPSL-CM6A-LR (DISO = 5.341) perform better in precipitation simulation, while CNRM-CM6-1 (DISO = 5.569) and CNRM-ESM2-1 (DISO = 5.570) show poorer performance. In terms of wind speed (WIN) simulation, UKESM1-0-LL shows the lowest DISO value of 0.876, indicating the best performance, while CNRM-ESM2–1 has the highest DISO value of 0.905, suggesting relatively large wind speed simulation errors. MPI-ESM1-2-HR (DISO = 0.902) performs slightly better than CNRM-ESM2–1 but still has relatively poor wind speed simulation capability. The MME (DISO = 0.878) ranks second, just behind UKESM1-0-LL. For relative humidity (RHU) simulation, MME has the lowest DISO value of 0.782, indicating the best simulation capability, while GFDL-ESM4 has the highest DISO value of 0.814, implying relatively large errors in simulating relative humidity. Additionally, UKESM1-0-LL (DISO = 0.793) and MPI-ESM1-2-HR (DISO = 0.794) perform relatively well in simulating this variable. For downward shortwave radiation (RSDS) simulation, the MME achieves the lowest DISO value of 0.196, representing the best performance, while CanESM5 records the highest DISO value of 0.209, indicating the poorest performance. IPSL-CM6A-LR and CNRM-CM6–1 have DISO values of 0.207 and 0.204, respectively, reflecting weaker performance for this variable, while MPI-ESM1-2-HR and GFDL-ESM4, both with a DISO of 0.200, demonstrate better capability in simulating this variable. For air pressure (PRS) simulation, MME has the highest DISO value of 0.693, showing the worst performance, while EC-Earth3 has the lowest DISO value of 0.679, indicating the best performance. GFDL-ESM4, with a DISO of 0.682, also demonstrates good capability in simulating air pressure.

Among the simulations of various meteorological variables, the multi-model ensemble (MME) demonstrates the most stable overall performance and shows strong simulation capability for several key variables (**Figure 2**). For precipitation (PRE) and relative humidity (RHU) simulations, the DISO values of MME are 4.480 and 0.782, respectively, the lowest among all models, indicating that it effectively reduces the systematic errors of individual models and improves the accuracy and stability of the simulations for these two variables. For temperature (TAV), wind speed (WIN), and downward shortwave radiation (RSDS), the DISO values of MME are 13.307, 0.878, and 0.196, respectively, ranking second in simulation capability, and it can reasonably capture the spatiotemporal distribution characteristics of these variables. However, for air pressure (PRS) simulation, the DISO value of MME is 0.693, representing the poorest performance. Although MME’s simulation capability for this variable is slightly inferior to that of individual models, its overall performance remains superior when considering all meteorological variables collectively.

**Figure 3:**
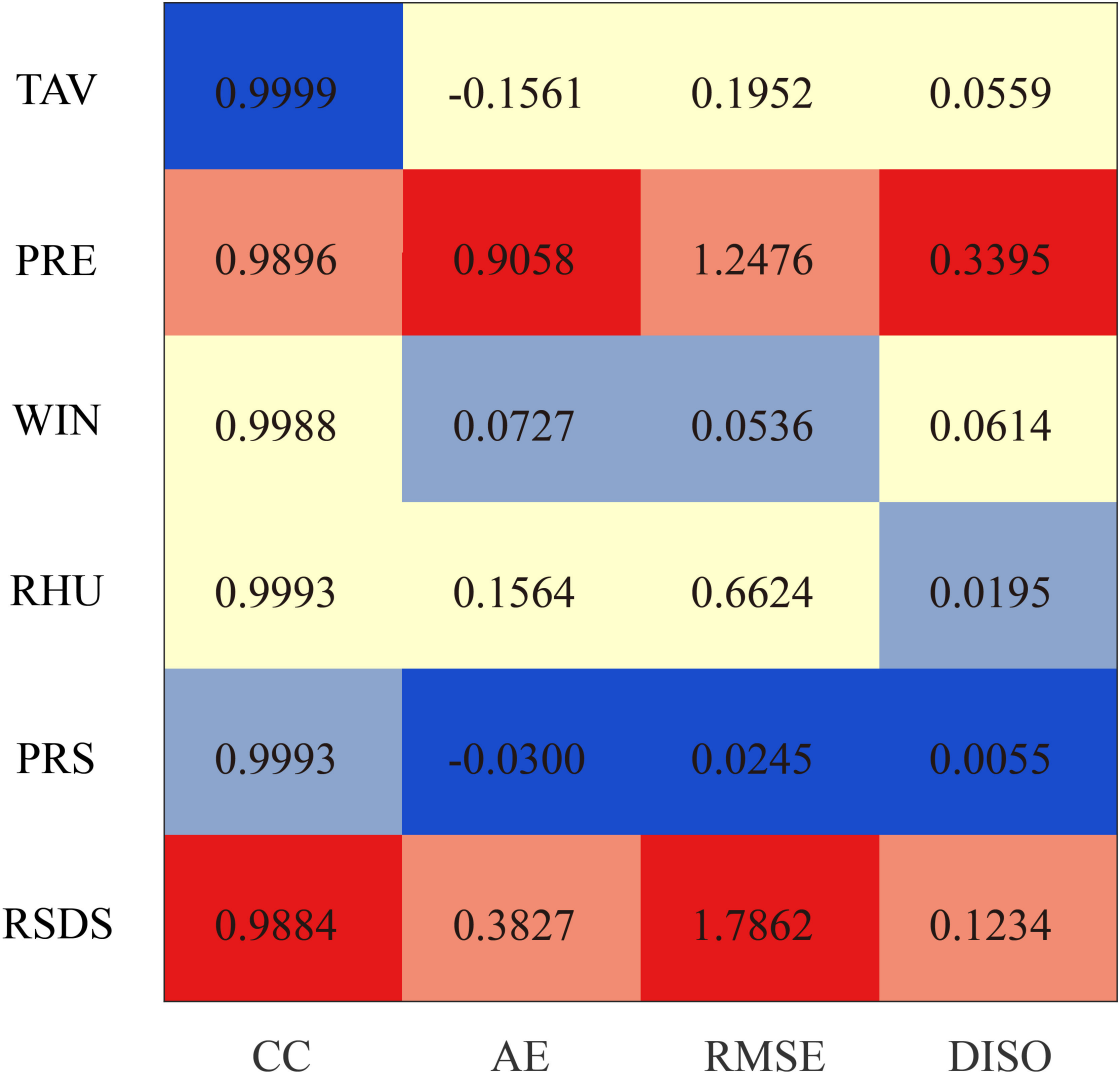
Correlation coefficient (CC), absolute error (AE), root mean square error (RMSE), and DISO values of high-resolution historical data (1961–2014) generated using the BCCAQ method.

Based on meteorological data from 105 stations in Xinjiang, we validated the high-resolution historical data (1961–2014) generated using the BCCAQ method and found that it exhibits higher correlations and lower errors in the simulation accuracy of multiple key meteorological variables, particularly showing significant advantages in variables such as temperature (TAV), wind speed (WIN), relative humidity (RHU), and pressure (PRS) (**Figure 3**). The correlation coefficients (CC) of the BCCAQ data for these variables are all close to 1, such as TAV (0.9999), WIN (0.9988), RHU (0.9993), and PRS (0.9993), all higher than those of MME, indicating that it has higher reliability in reproducing the variation trends of meteorological elements. Meanwhile, the Absolute Error (AE) and Root Mean Square Error (RMSE) of the BCCAQ data are significantly reduced. For example, the AE and RMSE of TAV are only −0.1561°C and 0.1952°C, which are far better than the values of 1.585°C and 4.703°C from MME, indicating the high accuracy of BCCAQ in temperature simulation. The DISO value for air pressure (PRS) is only 0.0055, which is significantly lower than MME’s 0.570, further confirming its advantage in meteorological variable simulation. Regarding precipitation (PRE) and downward shortwave radiation (RSDS), the AE, RMSE, and DISO values of BCCAQ are consistently lower than those of MME. For instance, PRE’s DISO is 0.3395, while MME’s is 0.753, demonstrating that BCCAQ provides greater accuracy in both precipitation and radiation simulations. Overall, the BCCAQ method significantly improves the simulation accuracy of meteorological elements, reduces model errors, and performs particularly well in key variables such as temperature, wind speed, relative humidity, and air pressure, providing more reliable high-resolution data support for climate change research in Xinjiang.

### 3.2 Spatiotemporal characteristics of meteorological variables in Xinjiang

This section analyzes the spatiotemporal characteristics of major meteorological variables in Xinjiang, focusing on temperature, precipitation, wind speed, relative humidity, downward shortwave radiation, and surface pressure. The comparison between the historical period (1991–2020) and future projections (2031–2060) highlights their trends and spatial heterogeneity, providing insights into regional climate evolution and hydroclimatic change.

#### 3.2.1 Temperature

**Figure 4:**
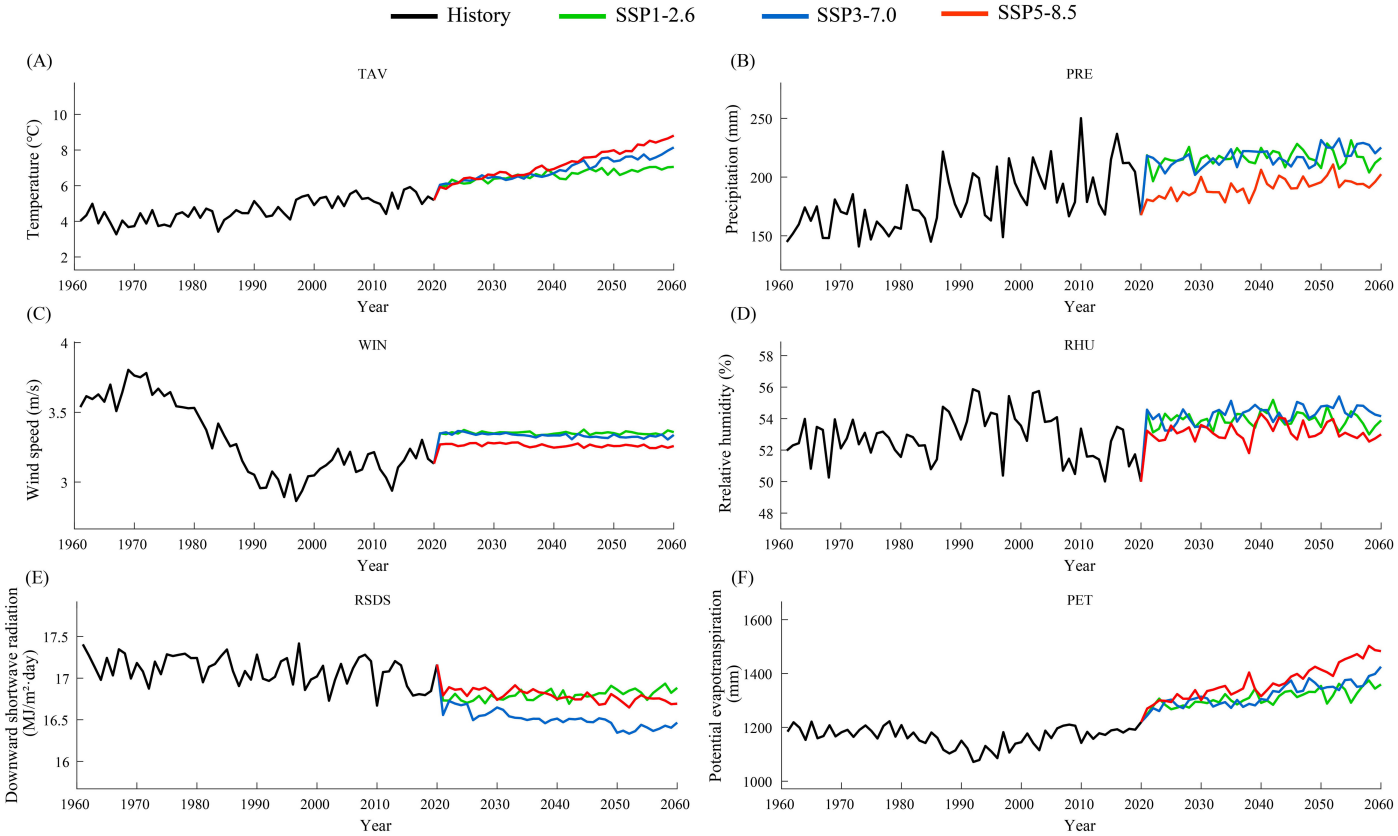
Temporal variation characteristics of temperature, precipitation, wind speed, relative humidity, downward shortwave radiation, and potential evapotranspiration **(A–F)** in Xinjiang from 1961 to 2060. The black solid line represents the time series of meteorological variables from 1961 to 2020. The green solid line represents the time series of meteorological variables from 2015 to 2060 under the SSP126 scenario, the blue solid line represents the SSP370 scenario, and the orange solid line represents the SSP585 scenario.

**Figure 5:**
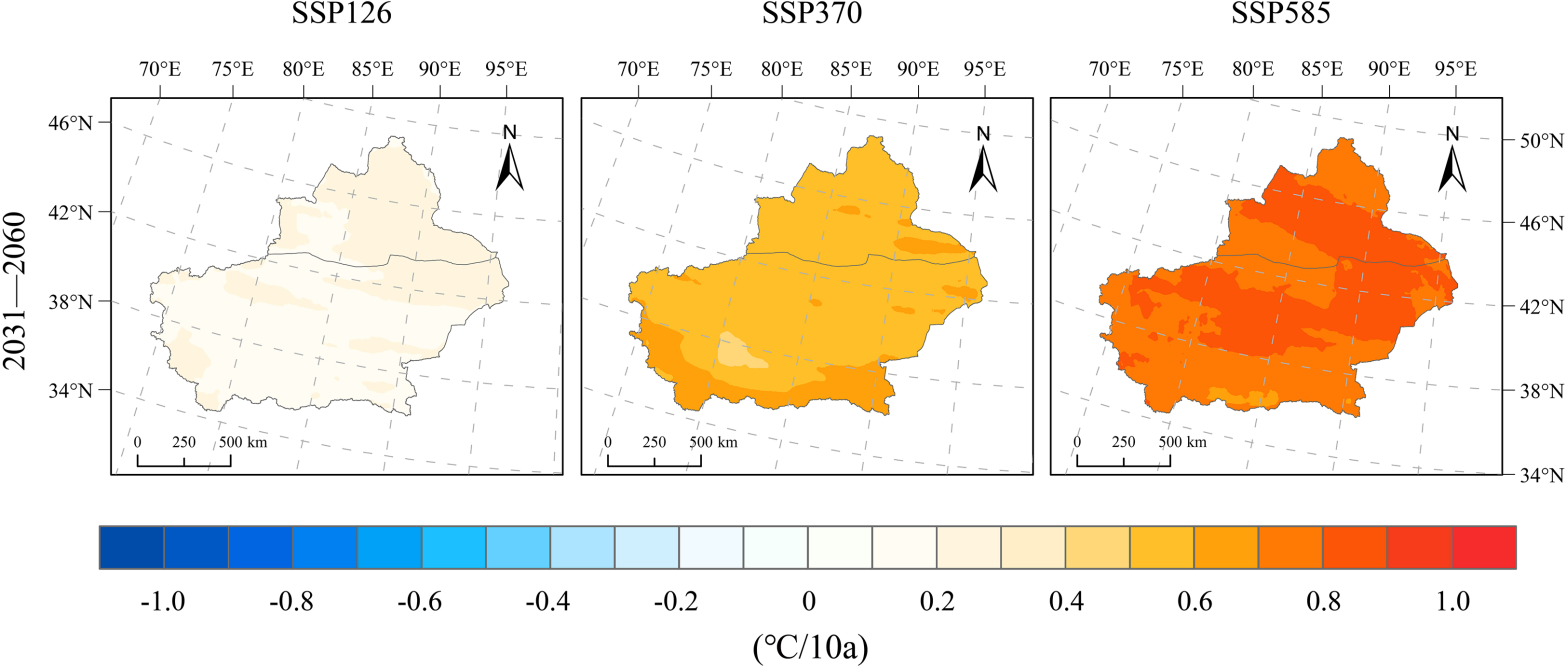
Spatial distribution of change rates for temperature changes under SSP126, SSP370, and SSP585 scenarios for 2031–2060 relative to 1991–2020.

The mean temperature in Xinjiang during 1991–2020 was 5.1°C, showing a slight upward trend (**Figure 4**), with an overall warming rate of 0.28°C/10a. After 2020, Xinjiang’s temperature is projected to increase continuously across various future scenarios (SSP126, SSP370, and SSP585). Under the SSP126 scenario, the temperature during 2031–2060 rises by 1.64°C compared with 1991–2020, with an average warming rate of 0.20°C/10a. Northern Xinjiang exhibits a notably higher warming rate compared with southern Xinjiang, with non-Tianshan regions in the north warming at 0.2–0.3°C/10a, whereas most parts of southern Xinjiang warm at 0.1–0.2°C/10a (**Figure 5**). Under the SSP370 scenario, the magnitude and rate of temperature increase in Xinjiang are noticeably larger (**Figures 4** and **5**). The temperature during 2031–2060 increases by 2.04°C relative to 1991–2020, with a warming rate of 0.58°C/10a. Most regions of Xinjiang warm at 0.5–0.6°C/10a, while the southern mountainous areas and parts of eastern northern Xinjiang experience higher warming rates of 0.6–0.7°C/10a (**Figure 5**).

#### 3.2.2 Precipitation

**Figure 6:**
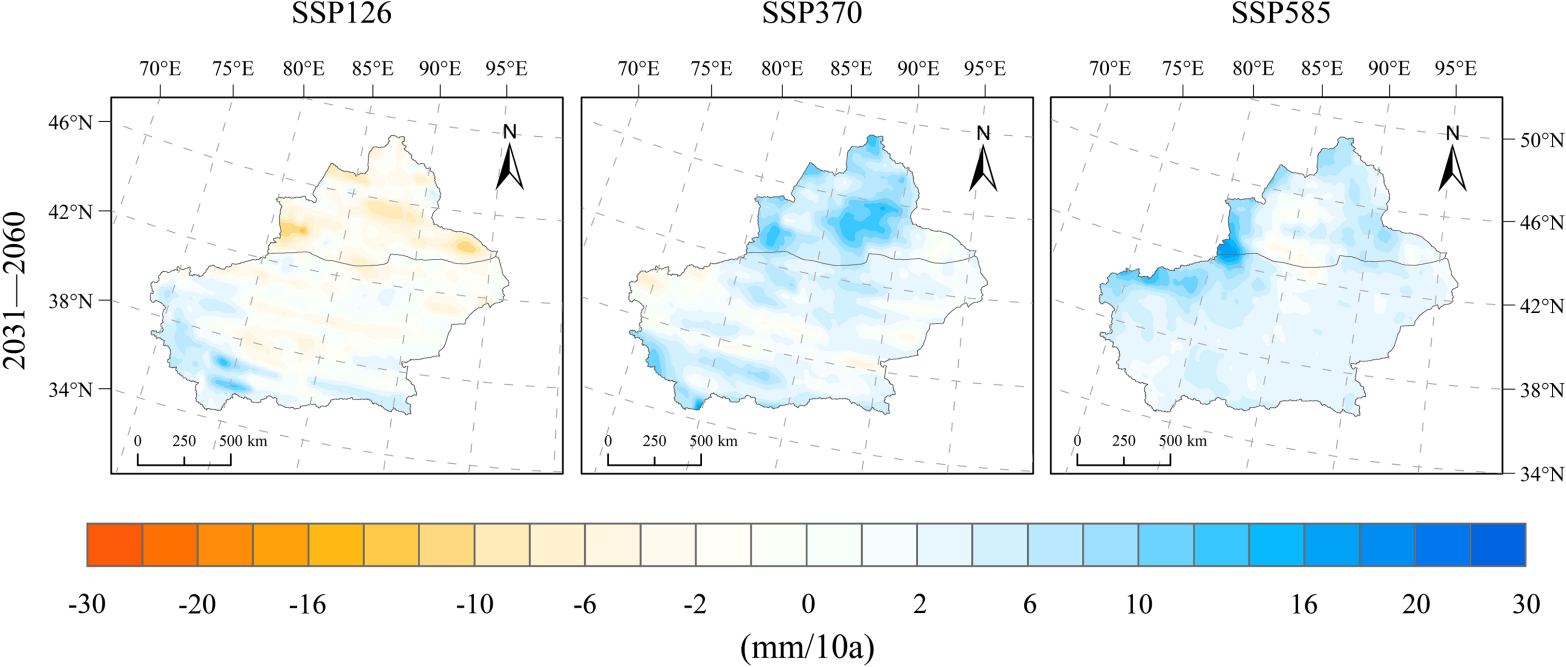
Spatial distribution of change rates for precipitation changes under SSP126, SSP370, and SSP585 scenarios for 2031–2060 relative to 1991–2020.

The average annual precipitation in Xinjiang during 1991–2020 was 193.8 mm, exhibiting an increasing trend over the 30-year period, with a regional mean growth rate of 5.07 mm/10a. Under future scenarios, precipitation in Xinjiang shows a slight increase after 2020; under the SSP126 scenario, there is no significant trend, while under SSP370 and SSP585, precipitation continues to increase (**Figure 4**). Under the SSP126 scenario, precipitation during 2031–2060 increases by 24.3 mm compared to 1991–2020, with a corresponding rate of change of −0.7 mm/10a. A significant decreasing trend is observed in northern Xinjiang, with the rate of decline ranging from −12–2 mm/10a in the basin areas. In southern Xinjiang, particularly in the southern and western parts, precipitation shows a slight increasing trend, with rates between 2 and 12 mm/10a. No clear trend is detected in the basin areas of southern Xinjiang. Under the SSP370 scenario, the precipitation during 2031–2060 increases by 26.3 mm compared to 1991–2020. The rate of change in precipitation over Xinjiang is 3.87 mm/10a under this scenario, with nearly the entire region exhibiting an increasing trend. Specifically, the precipitation trend in northern Xinjiang ranges from 4–14 mm/10a, while in southern Xinjiang it ranges from −2–6 mm/10a (**Figure 6**). The SSP585 scenario represents the case with the smallest change in total precipitation but the highest rate of increase. The precipitation amount in 2031–2060 drops slightly by 0.04 mm relative to the period 1991–2020. The overall rate of change across Xinjiang is 3.93 mm/10a. In northern Xinjiang, the change rate ranges from −1–6 mm/10a. The central basin shows no significant trend. The eastern region exhibits an upward trend with a rate above 0.4 mm/10a. In eastern southern Xinjiang, precipitation rises slowly, at a rate of 2–4 mm/10a. In the western part, the rate ranges from 1–10 mm/10a, with the highest values concentrated in the western Tianshan region (**Figure 6**).

#### 3.2.3 Wind Speed

**Figure 7:**
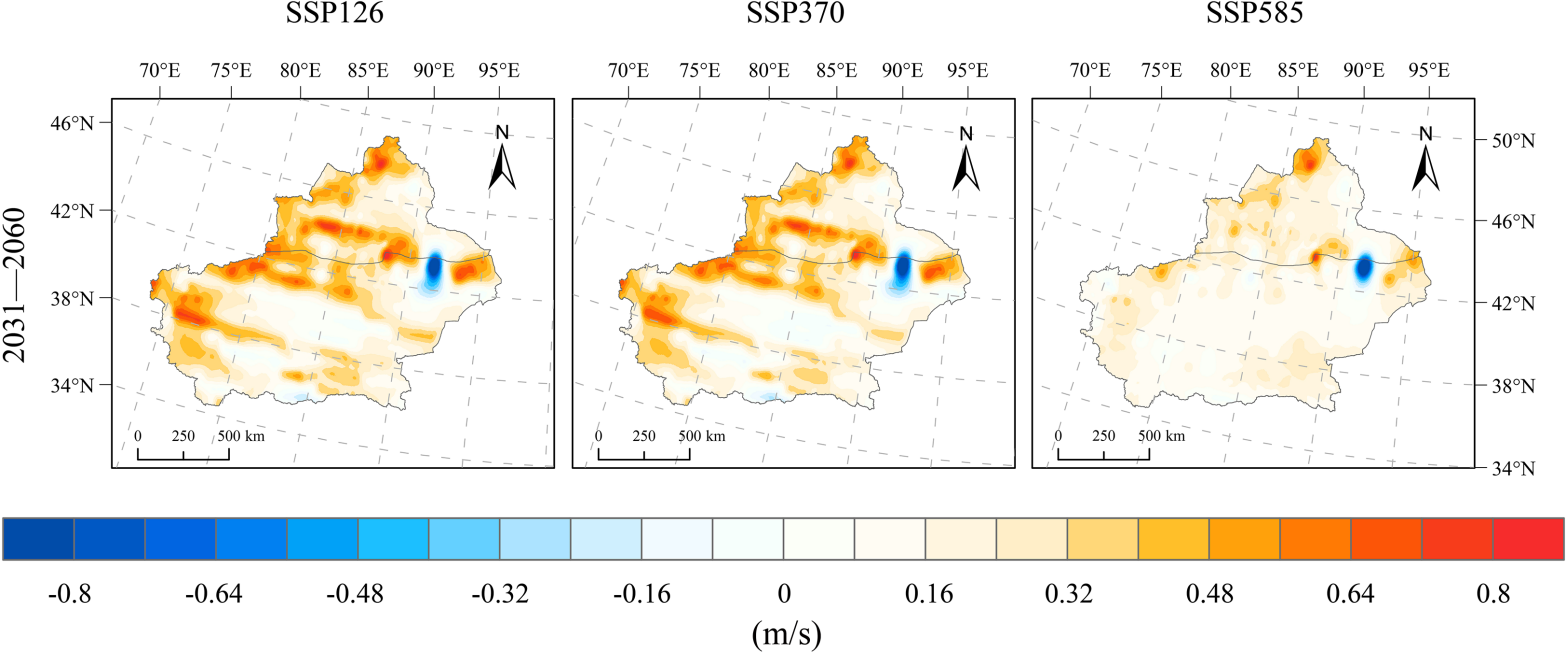
Spatial distribution of wind speed changes under SSP126, SSP370, and SSP585 scenarios for 2031–2060 relative to 1991–2020.

The average wind speed in Xinjiang from 1991 to 2020 was 3.09 m/s, showing an upward trend, with a regional mean increase rate of 0.07 m/s per decade. Under future scenarios (SSP126, SSP370, and SSP585), wind speed in Xinjiang shows no significant variation after 2020, remaining stable at 3.2–3.4 m/s. No significant differences are observed between the scenarios. Relative to the historical period (1991–2020), the wind speed during 2031–2060 is projected to increase by 0.26 m/s under the SSP126 scenario, 0.24 m/s under SSP370, and 0.17 m/s under SSP585 (**Figure 4**). From the spatial distribution of wind speed changes, Xinjiang generally exhibits an increasing wind speed trend during 2031–2060 under the SSP126 scenario, except for a notable decline in the area near Shisanjianfang, where the change ranges from −1.2–−0.4 m/s, while other regions experience increases of 0.1–0.8 m/s (**Figure 7**). The wind speed increases are more pronounced in the mountainous regions of Xinjiang, with western mountains and the northern Tianshan area experiencing increases exceeding 0.4 m/s, reaching up to 0.8 m/s. The spatial patterns of wind speed changes under the SSP370 and SSP585 scenarios are nearly identical to those under SSP126 during the same period, indicating limited impact of different emission scenarios on wind speed. Wind speed variations under SSP370 closely align with those under SSP126. Wind speed increases under SSP585 are notably weaker across Xinjiang compared to SSP126 and SSP370. With the exception of the Shisanjianfang region, wind speed in southern Xinjiang increases by 0.08–0.24 m/s, while northern Xinjiang shows more significant increases between 0.16 and 0.48 m/s, reaching up to 0.8 m/s in the western Altai Mountains.

#### 3.2.4 Relative humidity

**Figure 8:**
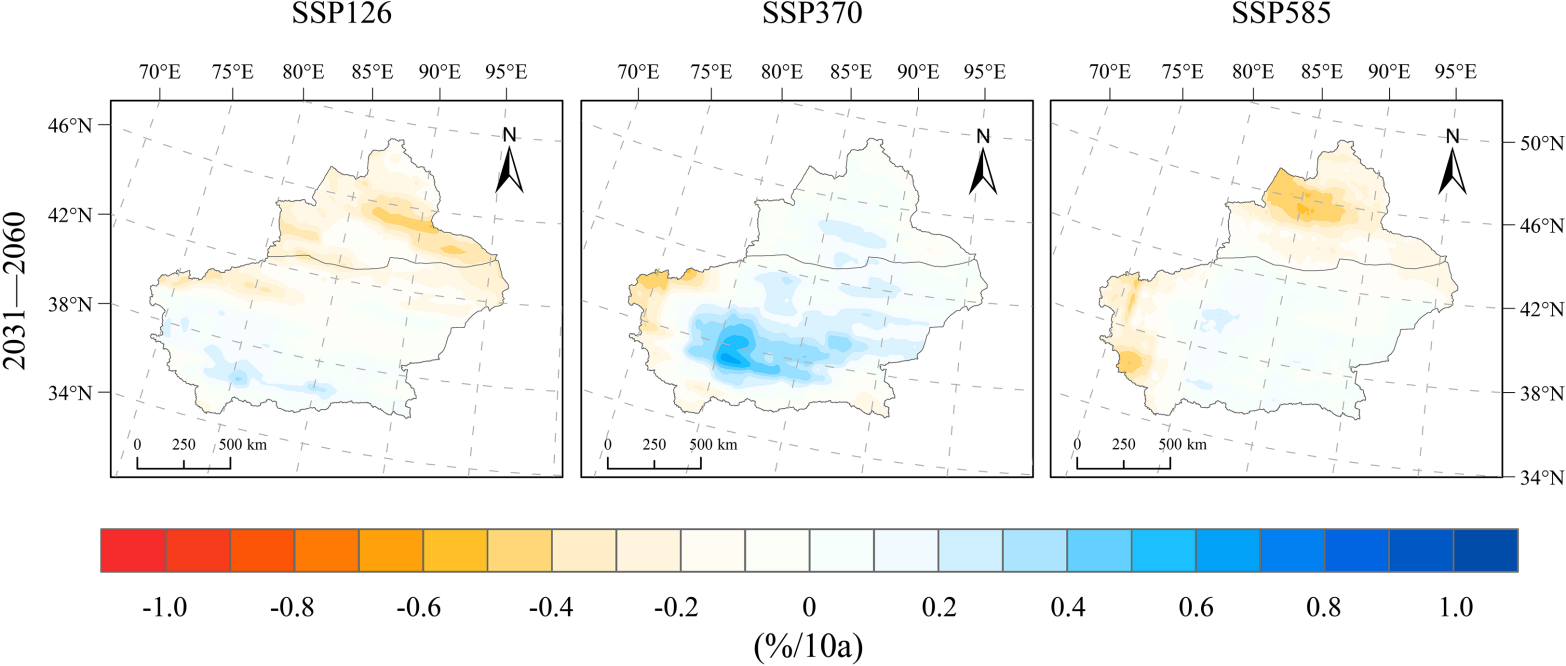
Spatial distribution of change rates for relative humidity changes under SSP126, SSP370, and SSP585 scenarios for 2031–2060 relative to 1991–2020.

From 1991 to 2020, the average relative humidity in Xinjiang was 52.98%, exhibiting a decreasing trend at a rate of −1.38%/10a. Under future scenarios (SSP126, SSP370, and SSP585), the relative humidity during 2031–2060 increases by 0.99%, 1.46%, and 0.21%, respectively, compared to 1991–2020. Regarding the temporal trend, changes in relative humidity across Xinjiang after 2020 remain relatively minor (**Figure 4**). For both SSP126 and SSP585, a drying trend dominates northern Xinjiang, whereas some regions in the south display a slight humidification trend. Under the SSP126 scenario, areas including and north of the Tianshan Mountains exhibit a drying trend with a rate of −0.6–−0.1%/10a, while areas south of the Tianshan Mountains display a slight moistening trend at a rate of 0–0.3%/10a (**Figure 8**). Under the SSP370 scenario, the change rate of relative humidity in Xinjiang is generally characterized by a moistening trend, with the basin regions mainly showing an increase ranging from 0.1%–0.8%/10a; southern Xinjiang exhibits a more pronounced moistening trend, while slight drying occurs in the mountainous areas of southern Xinjiang at a rate of −0.6%–−0.2%/10a, and no significant changes are observed in other regions. Under the SSP585 scenario, a drying trend is observed in the northern Xinjiang basin and western parts of southern Xinjiang, with rates ranging from −0.7%–−0.3%/10a; other areas in northern Xinjiang exhibit a slight drying trend at rates of −0.3%–0%/10a, while the southern Xinjiang basin shows a slight moistening trend with a change rate of 0%–0.3%/10a.

#### 3.2.5 Downward shortwave radiation

**Figure 9:**
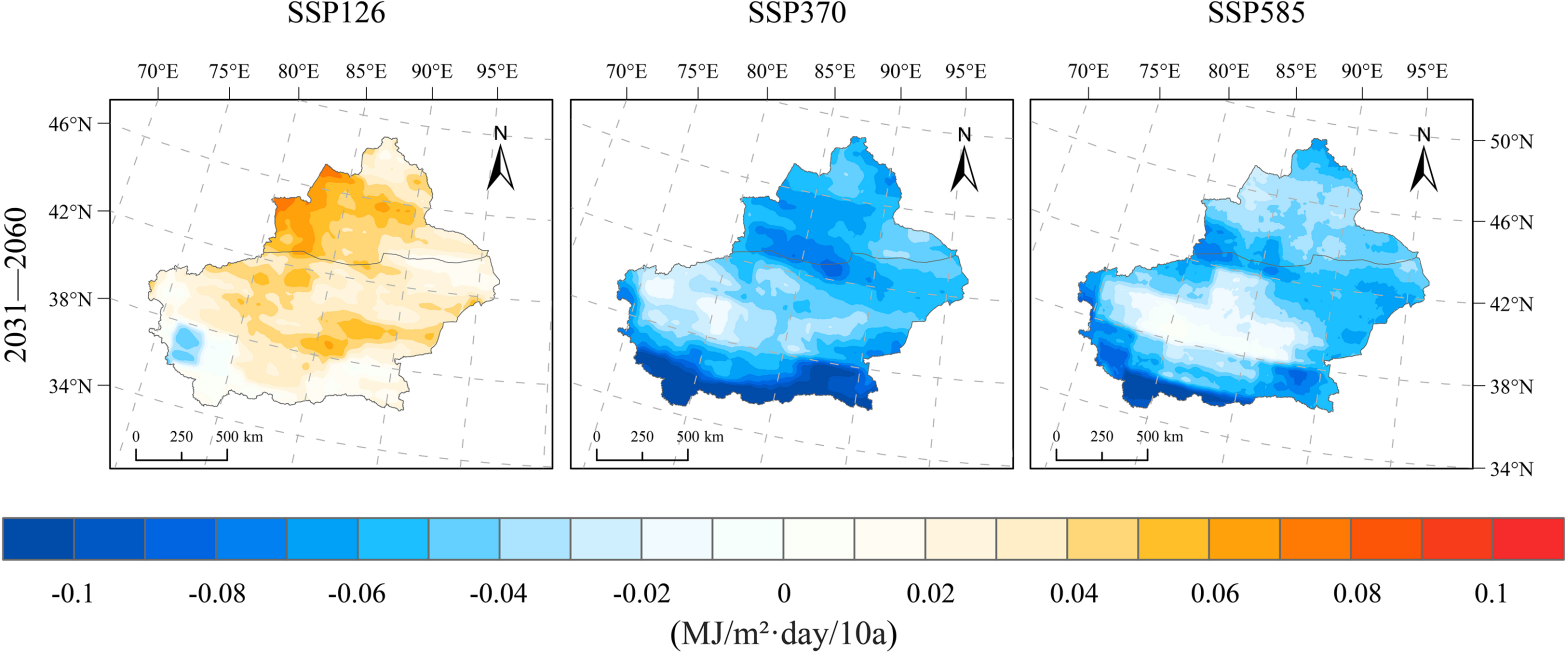
Spatial distribution of change rates for downward shortwave radiation changes under SSP126, SSP370, and SSP585 scenarios for 2031–2060 relative to 1991–2020.

From 1991 to 2020, the average downward shortwave radiation in Xinjiang was 17.03 MJ/m²·day, showing a declining trend over the past 30 years with a change rate of −0.04 MJ/m²·day/10a. The projected changes in downward shortwave radiation across Xinjiang after 2020 vary among the future emission scenarios (SSP126, SSP370, and SSP585). Under the SSP126 scenario, the radiation during 2031–2060 decreases by 0.219 MJ/m²·day compared to 1991–2020, with a change rate of 0.033 MJ/m²·day/10a (**Figure 4**). Throughout Xinjiang, radiation shows an upward trend during 2031–2060, with the exception of the southwestern mountainous region, where increases range between 0.01 and 0.08 MJ/m²·day/10a. The upward trend is especially prominent in the northern Xinjiang basin, where radiation increases by 0.04–0.08 MJ/m²·day/10a. A slight declining trend in radiation is observed in the southwestern mountainous areas of southern Xinjiang, with rates ranging from −0.05–0 MJ/m²·day/10a (**Figure 9**). Under the SSP370 scenario, future downward shortwave radiation in Xinjiang is projected to decrease significantly compared to 1991–2020, with a magnitude of −0.568 MJ/m²·day and a declining trend of −0.058 MJ/m²·day/10a. Spatially, a clear declining trend in radiation is observed throughout Xinjiang, with the decrease more prominent in mountainous areas than in basins. In southern Xinjiang’s mountainous regions, the decline is greatest (−0.15–−0.06 MJ/m²·day·10a), while in southern basins, it is more moderate (−0.05–−0.01 MJ/m²·day·10a). Under the SSP585 scenario, future downward shortwave radiation in Xinjiang also shows a slight decline relative to 1991–2020, with a reduction of −0.26 MJ/m²·day and a trend of −0.046 MJ/m²·day/10a. The spatial distribution of the trend mirrors that of the SSP370 scenario but with less pronounced reductions. The decrease in southern Xinjiang’s mountainous areas ranges from −0.12–−0.05 MJ/m²·day·10a, whereas in the basin areas, the reduction is milder, with rates between −0.04 and 0.01 MJ/m²·day·10a.

#### 3.2.6 Potential evapotranspiration

**Figure 10:**
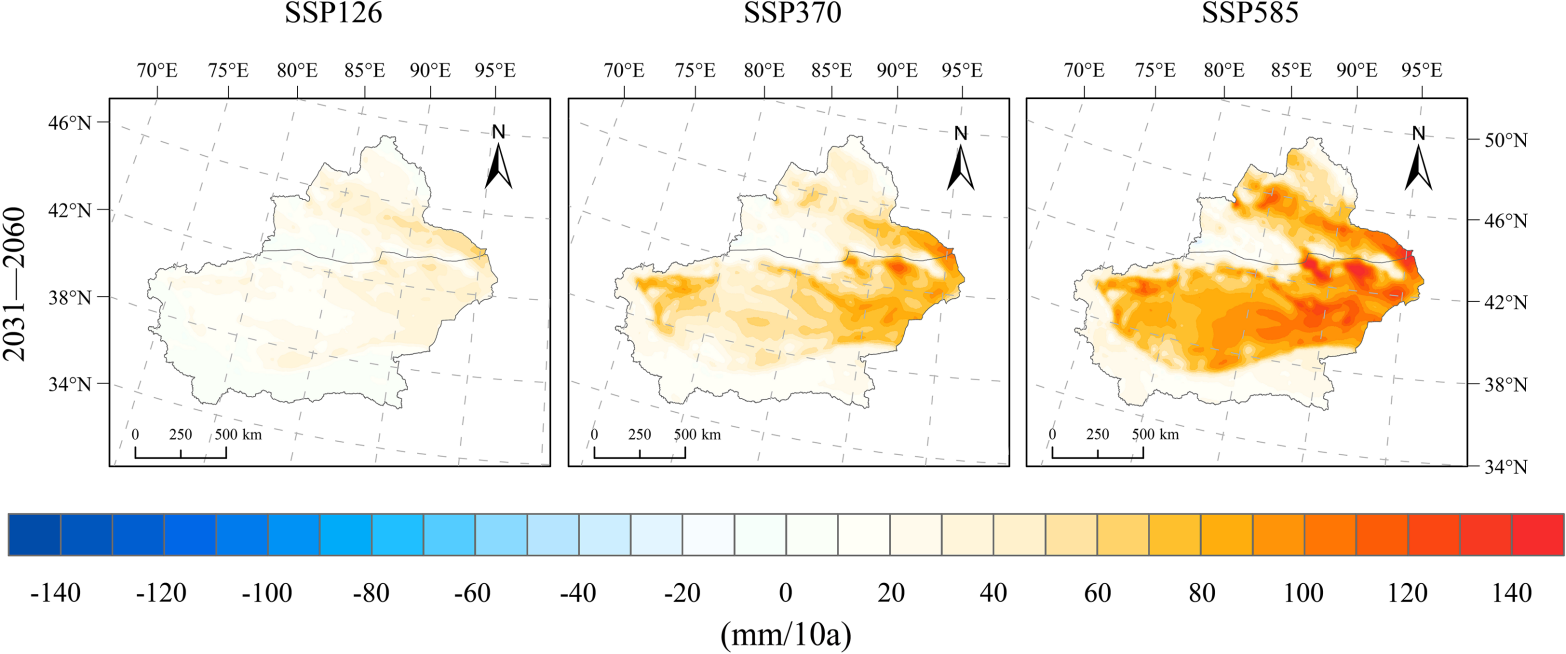
Spatial distribution of change rates for potential evapotranspiration changes under SSP126, SSP370, and SSP585 scenarios for 2031–2060 relative to 1991–2020.

From 1991 to 2020, the average potential evapotranspiration (PET) in Xinjiang was 1159.0 mm, with an average increasing trend of 35.8 mm/10a. Following 2020, PET in Xinjiang shows an increasing trend under all future scenarios (SSP126, SSP370, and SSP585), with SSP126 exhibiting a modest rise and the other two scenarios showing stronger upward trends. Under SSP126, PET for 2031–2060 increases by 205.1 mm relative to 1991–2020, corresponding to a growth rate of 21.5 mm/10a. From 2031 to 2060, the PET increase in Xinjiang’s mountainous areas is relatively small, at 0–10 mm/10a, while most basin regions exhibit significant increases ranging from 10–50 mm/10a. PET increases more rapidly in the eastern part of the region, with areas around Tianchi and Naomao Lake exhibiting the highest rates, up to 60 mm/10a (**Figure 10**). Under the SSP370 scenario, PET during 2031–2060 increases by 219.6 mm compared to 1991–2020, with a change rate of 42.9 mm/10a. The increasing trend of PET in this scenario is significantly greater than that under the SSP126 scenario. Specifically, PET in eastern Xinjiang and the southern basin areas increases at a rate of 30–110 mm/10a. PET increases even more significantly under the SSP585 scenario, with a 313.5 mm rise from 1991–2020 to 2031–2060, reflecting a rate of 63.3 mm/10a. More specifically, PET in the mountainous areas of Xinjiang shows a slight increase (0–40 mm/10a), whereas a pronounced increase is observed in the basin regions (40–110 mm/10a), peaking near Tianchi at 160 mm/10a.

### 3.3 Spatiotemporal characteristics of dry–wet variability in Xinjiang

**Figure 11:**
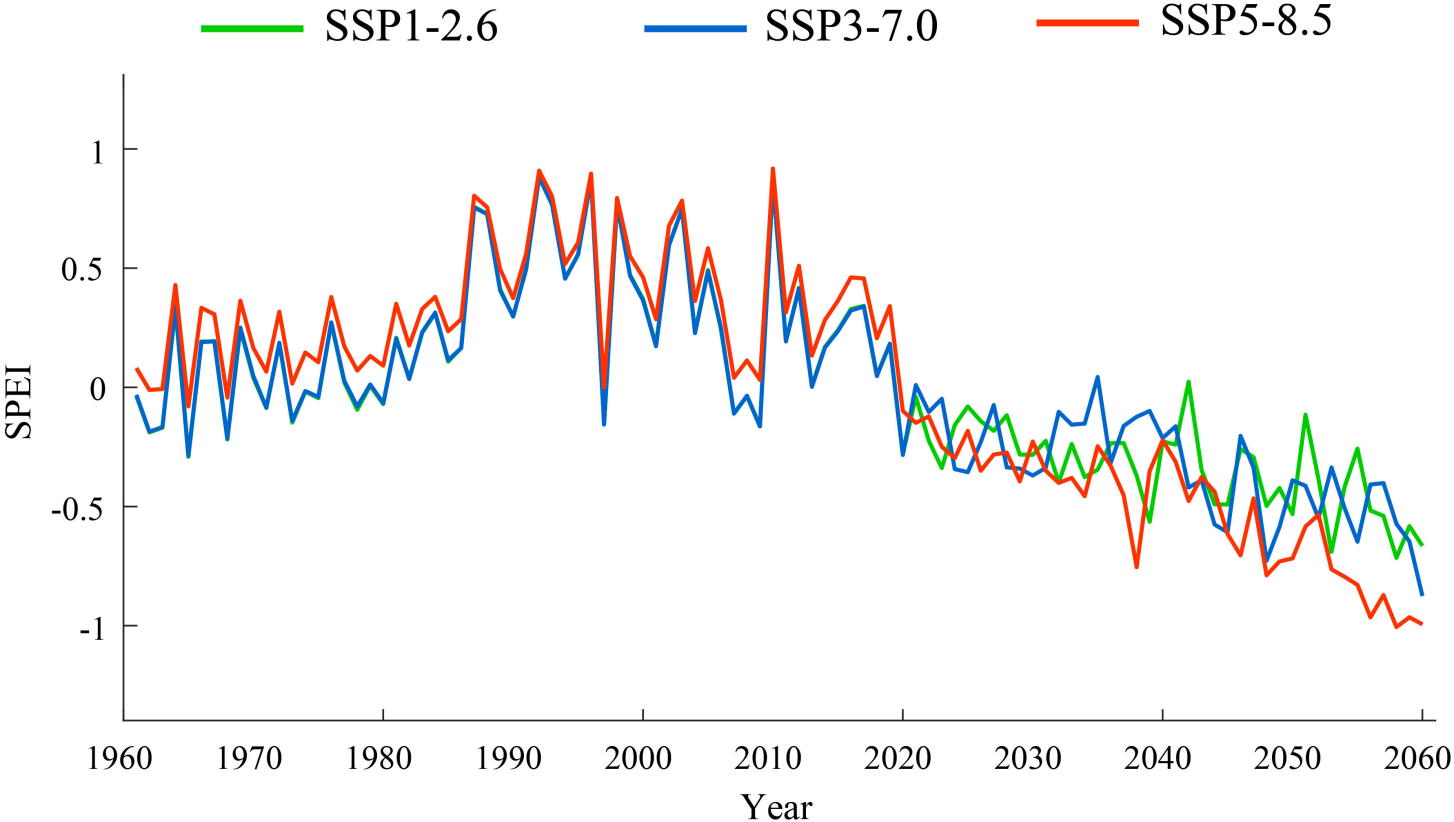
Temporal variations of SPEI under SSP126, SSP370, and SSP585 scenarios from 1961 to 2060.

This study investigates the spatiotemporal characteristics of SPEI under future scenarios (SSP126, SSP370, and SSP585). Under the SSP126 scenario, the mean SPEI in Xinjiang was 0.3397 during 1991–2020, and is projected to decline to −0.3883 during 2031–2060, indicating drier conditions (**Figure 11**). The average SPEI trend in Xinjiang was −0.215/10a during 1991–2020, and is projected to be −0.112/10a during 2031–2060. In the SSP370 scenario, the average SPEI in Xinjiang during 1991–2020 was 0.3379, decreasing to −0.3792 in 2031–2060, indicating more pronounced aridity. The average SPEI trend during 1991–2020 was −0.215/10a, and is projected to be −0.181/10a in 2031–2060. Under the SSP585 scenario, the average SPEI in Xinjiang was 0.4410 during 1991–2020, decreasing sharply to −0.8099 in 2031–2060, indicating significantly drier conditions. The trend of SPEI during 1991–2020 was −0.175/10a, which intensifies to −0.236/10a in 2031–2060.

**Figure 12:**
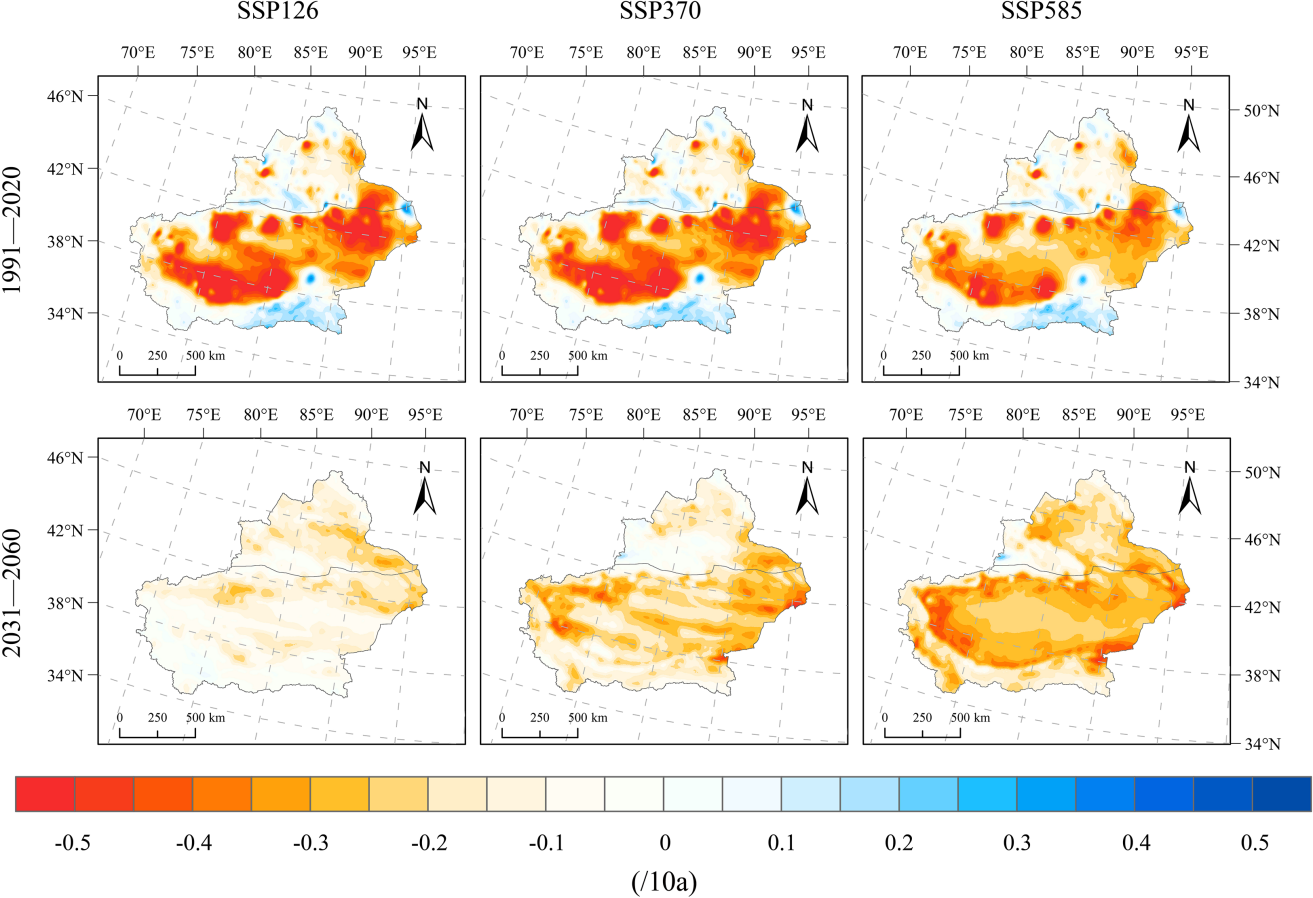
Spatial distribution of SPEI change rates under SSP126, SSP370, and SSP585 scenarios for 1991–2020 and 2031–2060.

The spatial pattern of SPEI change rates during the historical period is largely consistent across the three scenarios, indicating severe aridification in the southern Xinjiang Basin, mild aridification in the northern basin, and localized humidification in parts of the mountainous areas of Xinjiang (**Figure 12**). During 1991–2020 under SSP126, Xinjiang exhibited a predominantly aridifying trend, with the SPEI decreasing at a rate of −0.7–−0.1/10a in most regions, while slight humidification occurred only in the eastern Kunlun and Tianshan mountain areas in southern Xinjiang. Specifically, stronger aridification was observed in the southern Xinjiang Basin and the eastern part of northern Xinjiang, with change rates ranging from −0.7–−0.3/10a; moderate aridification occurred in other parts of the southern basin (−0.3–−0.1/10a), while the eastern Kunlun and Tianshan mountainous regions exhibited a humidification trend (0.05–0.2/10a). During 2031–2060, Xinjiang is expected to continue aridifying, with drier conditions than in 1991–2020. However, the aridification trend slightly weakens, especially in basin regions where SPEI decreases at rates of −0.25–−0.1/10a. Aridification is less pronounced in Xinjiang’s mountainous regions (−0.05–0/10a), while the southern and northern basins show clear drying trends (−0.2–−0.05/10a), and the eastern region undergoes the strongest aridification, with SPEI decreasing at rates of −0.3–−0.15/10a. From 1991 to 2020, Xinjiang showed a dominant drying trend under SSP370, mirroring the spatial distribution observed in SSP126. The aridification zones were primarily found in the southern basin and eastern parts of Xinjiang, with SPEI change rates between −0.7 and −0.25 per decade, whereas humidification occurred mainly in the northern basin and eastern Kunlun Mountain region, with rates of 0.1–0.25/10a. During 2031–2060, an aridification trend is projected across the entire Xinjiang region, resulting in drier conditions compared to the historical period. Aridification is most pronounced in the southern basin and eastern Xinjiang, with SPEI declining at −0.35–−0.15/10a, whereas mountain regions including the Tianshan, Kunlun, and Altai ranges exhibit milder drying trends (−0.1–−0.05/10a). During 1991–2020, the SSP585 scenario showed a mainly aridifying trend in Xinjiang, resembling the pattern observed in SSP126. The drying areas in the southern basin and eastern Xinjiang had SPEI change rates of −0.7–−0.2/10a, whereas humidification in northern Xinjiang and eastern Kunlun showed rates of 0–0.2/10a. During 2031–2060, persistent drying is anticipated across Xinjiang, with SPEI declining at −0.4–−0.2/10a in most areas. The drying trend is more intense in the southern region than in the north and more severe in basin areas compared to mountainous regions. Specifically, the mountainous regions of Xinjiang show a weaker aridification trend, with SPEI changing at −0.2–−0.1/10a; the northern Xinjiang Basin has a change rate of −0.3–−0.15/10a, whereas the southern Xinjiang Basin is more severely affected, with the western part experiencing a rate of −0.4–−0.2/10a.

## 4 Discussion

This research provides substantial scientific insight into data construction and the depiction of hydroclimatic variations. We applied a BCCAQ-based bias correction method, using ERA5 reanalysis and station observations as reference, to systematically adjust the daily multivariate CMIP6 simulation data over Xinjiang. The simulation accuracy of key meteorological variables such as temperature, wind speed, relative humidity, and atmospheric pressure was significantly improved. The method exhibited notable advantages in areas characterized by rugged terrain and strong climatic heterogeneity, validating its effectiveness for climate reconstruction in arid regions. These results offer empirical support for the application of statistical downscaling methods in high-altitude arid regions. Additionally, this study establishes a critical data basis for precise future climate assessments and modeling of extreme events.

From a supply–demand perspective of meteorological drivers, this study demonstrates the dominant role of temperature and potential evapotranspiration in shaping future drought evolution, highlighting the critical “warming–enhanced evapotranspiration–intensified drought” pathway. This mechanism is consistent with global assessments of intensifying drought, where rising temperatures have been shown to markedly accelerate drought severity (Zeng et al., 2023; Gebrechorkos et al., 2025). Moreover, spatiotemporal variations in evapotranspiration components during drought periods have been identified as key regulators of land–atmosphere interactions (Zhang K. et al., 2024). Under the SSP585 high-emission scenario, drought intensity increases substantially, with the Tarim Basin and eastern Xinjiang exhibiting a pronounced warming–drying trend. This pattern echoes findings across Central Asia, where modest precipitation gains are outweighed by the rapid escalation of potential evapotranspiration, making it the decisive driver of rising drought risk.

Previous studies have shown that precipitation has increased in northern Xinjiang and mountainous regions in recent decades, whereas the Tarim Basin in southern Xinjiang has continued to experience persistent drying (Zhang Y. et al., 2024; Wang et al., 2025). Our results further confirm that Xinjiang is projected to exhibit a distinct hydroclimatic divergence, characterized by a “wet north–dry south, intensified aridity in basins, and relatively moderated changes in mountainous areas,” underscoring the crucial regulatory role of regional topography and climatic settings. Such a pattern not only constrains vegetation adaptation but also has the potential to reshape cropping systems and irrigation demands. With the intensification of warming and drying trends, vegetation communities in southern Xinjiang are likely to face degradation risks, whereas improved hydrothermal conditions in the north may favor pasture growth and crop production (Gu et al., 2025; Jing et al., 2025).

In contrast to studies such as Du et al. (2021) that primarily focused on precipitation simulations (Du et al., 2021), this work achieves advances in both the integration of multiple meteorological factors and the accuracy of data representation. These improvements enhance the understanding of Xinjiang’s climate change characteristics and drought-driving mechanisms, while also providing a robust methodological basis for quantitative assessments. Moreover, the findings carry significant implications for agricultural and water resource management. In the irrigation-dependent southern Xinjiang, the rapidly increasing evapotranspiration demand must be carefully considered to prevent overexploitation of scarce water resources. Conversely, in northern Xinjiang, the potential improvement in hydrothermal conditions may open opportunities for agricultural expansion, provided that such development is balanced with the carrying capacity of local soil and water resources to ensure long-term sustainability.

Nevertheless, this study has certain limitations. The ISIMIP bias-corrected dataset employed here has a spatial resolution of 0.5° × 0.5°, which is insufficient to fully resolve the fine-scale heterogeneity of Xinjiang’s mountainous–basin terrain, thereby constraining the spatial accuracy of the downscaled results (Xu et al., 2021; Rettie et al., 2023). In particular, the precipitation projections for 2031–2060 display strip-like spatial artifacts, which may stem from deficiencies in the representation of physical processes in the driving GCMs or from elevation-related interpolation errors. Furthermore, although the BCCAQ method improves the distributional characteristics and temporal structures of climate variables, its reliance on the stationarity of historical bias patterns introduces inherent structural uncertainties that remain difficult to eliminate (Rettie et al., 2023; Yang and Tang, 2023). Additional errors may also arise during interpolation to station locations, particularly in areas with complex topography (Xu et al., 2025).

Future research should further advance by employing regional climate models (RCMs), such as WRF, for dynamical downscaling. When nested within BCCAQ-corrected GCM outputs, these approaches offer the potential to more accurately capture Xinjiang’s complex topographic climate processes, particularly orographic precipitation and mountain wind systems. It is also essential to strengthen the detection and simulation of extreme climate events. Comprehensive assessments of the evolution and triggering mechanisms of extreme heat, heavy rainfall, and drought–wetness transitions in Xinjiang—supported by CMIP6 multi-model ensembles and high-resolution downscaling products—will improve predictive skill and provide deeper insights into their implications for socio-economic systems and potential adaptation pathways (Zhang B. et al., 2024). In addition, future studies should place greater emphasis on ecosystem responses to drought stress, with particular attention to changes in vegetation productivity, crop phenology, and the sustainability of artificial oasis systems. Such efforts are crucial to supporting ecological adaptation, advancing agricultural development, and optimizing water resource allocation in arid regions.

## 5 Conclusion

Based on multi-model ensemble outputs from CMIP6 and using the BCCAQ bias correction approach, this research systematically evaluates the projected changes in meteorological variables and spatiotemporal dry-wet trends in Xinjiang during 2031–2060 under three representative emission scenarios: SSP126, SSP370, and SSP585.The findings indicate that Xinjiang will undergo notable warming, enhanced potential evapotranspiration, and exacerbated aridification under global warming, with meteorological elements responding differently in both spatial pattern and intensity under various scenarios. The main conclusions are as follows:

1. The projected warming rates in Xinjiang for the period 2031–2060 are 0.20°C/10a (SSP126), 0.58°C/10a (SSP370), and 0.76°C/10a (SSP585), with warming intensifying under higher emission pathways. Spatially, the temperature increase is more pronounced in basins than in mountainous areas, and the north–south contrast is evident.
2. In the SSP126 scenario, changes in precipitation are insignificant, with slight reductions observed in the northern Xinjiang basin; in contrast, under SSP370 and SSP585, precipitation increases continuously, with rates above 3.8 mm/10a, especially in northern Xinjiang and the Tianshan region.
3. Wind speeds in 2031–2060 show an increase of 0.17–0.26 m/s compared to 1991–2020, with negligible inter-scenario variation and stable overall trends; the most notable increases occur in western mountainous regions and northern Tianshan, whereas the basins exhibit minor changes.
4. Relative humidity during 2031–2060 changes only slightly (0.21–0.46%) and remains relatively stable overall; SSP370 shows the most evident wetting trend, particularly in basins, whereas northern Xinjiang becomes drier under SSP126 and SSP585, with localized moistening in southern Xinjiang.
5. Downward shortwave radiation declines by −0.058 and −0.046 MJ/m²·day·10a under SSP370 and SSP585, respectively, with more pronounced decreases in mountainous areas than in basins, and the largest reduction observed in the mountainous regions of southern Xinjiang; under SSP126, a slight increasing trend is projected.
6. Potential evapotranspiration (PET) exhibits a consistent upward trend across the three scenarios, with change rates of 21.5, 42.9, and 63.3 mm/10a under SSP126, SSP370, and SSP585, respectively; the trend strengthens with emission intensity, especially in basins and eastern Xinjiang, whereas mountainous areas experience smaller increases.
7. The SPEI shows decreasing trends of −0.112/10a (SSP126), −0.181/10a (SSP370), and −0.236/10a (SSP585), indicating an increasing risk of aridification across Xinjiang. The Tarim Basin in southern Xinjiang and the eastern part of the region emerge as high‐risk hotspots, with drought severity intensifying markedly under higher emission pathways.

## Data Availability

The original contributions presented in the study are included in the article/supplementary material, further inquiries can be directed to the corresponding author/s.
